# Supplementary material for: Angiostrongylus cantonensis an Atypical Presenilin: Epitope Mapping, Characterization, and Development of an ELISA Peptide Assay for Specific Diagnostic of Angiostrongyliasis
Source: Membranes (Basel). 2022 Jan 19;12(2):108. doi: 10.3390/membranes12020108 (PMC8878667; doi:10.3390/membranes12020108)
Supplement: Supplementary file 1 [file membranes-12-00108-s001.zip › membranes-1532681-supplementary.pdf]

Supplementary Materials

Angiostrongilus cantonensis an Atypical Presenilin: Epitope Mapping, Characterization, and Development of an ELISA Peptide Assay for Specific Diagnostic of Angiostrongyliasis

Salvatore G. De-Simone [1,2,3,*], Paloma Napoleão-Pêgo [1], Priscila S. Gonçalves [1,3], Guilherme C. Lechuga [1], Arnaldo Mandonado, Jr. [4], Carlos Graeff-Teixeira [5] and David W. Provance, Jr. [1]

[1] Center of Technological Development in Health (CDTS), National Institute of Science and Technology for Innovation on Neglected Diseases (INCT-IDN), FIOCRUZ, Rio de Janeiro, RJ 21040-900, Brazil; paloma.pego@cdts.fiocruz.br (P.N.-P.); priscilla.desimone@cdts.fiocruz.br (P.S.G.); guilherme.lechuga@cdts.fiocruz.br (G.C.L.); bill.provance@cdts.fiocruz.br (D.W.P.J.)

[2] Laboratory of Epidemiology and Molecular Systematics (LESM), Oswaldo Cruz Institute, FIOCRUZ, Rio de Janeiro, RJ 21040-900, Brazil

[3] Department of Cellular and Molecular Biology, Biology Institute, Federal Fluminense University, Niterói, RJ 24220-900, Brazil

[4] Laboratory of Biology and Parasitology of Wild Mammals Reservoirs, Oswaldo Cruz Institute, FIOCRUZ, Rio de Janeiro, RJ 21040-360, Brazil; maldonado@ioc.fiocruz.br

[5] Infectious Diseases Unit, Department of Pathology, Federal University of Espirito Santo, Vitória, ES 29075-910, Brazil; carlos.teixeira@ufes.br

* Correspondence: dsimone@cdts.fiocruz.br

Spot	Sequence
A01	IHGRFVMEDGSGSNT
A02	VMEDGSGSNTDLKQE
A03	SGSNTDLKQEVKTVN
A04	DLKQEVKTVNDETAS
A05	VKTVNDETASKSKER
A06	DETASKSKERTISTT
A07	KSKERTISTTARNSK
A08	TISTTARNSKAASTN
A09	ARNSKAASTNGISEK
A10	AASTNGISEKVKRLY
A11	GISEKVKRLYGPTDM
A12	VKRLYGPTDMVKVFV
A13	GPTDMVKVFVPVSIT
A14	VKVFVPVSITMFIVV
A15	PVSITMFIVVTCVRN
A16	MFIVVTCVRNFDIYH
A17	TCVRNFDIYHGVNLI
A18	FDIYHGVNLIPTPYV
A19	GVNLIPTPYVIYNEP
A20	PTPYVIYNEPAADPG
A21	IYNEPAADPGTKLLH
A22	AADPGTKLLHAVANA
A23	TKLLHAVANAATFLV
A24	AVANAATFLVVVAIS
A25	ATFLVVVAISTFALL
A26	VVAISTFALLCLFYY
A27	TFALLCLFYYKFYCF
A28	CLFYYKFYCFLTGFI
A29	KFYCFLTGFIMFSTF
A30	LTGFIMFSTFLLVSL
B01	MFSTFLLVSLFSFVQ
B02	LLVSLFSFVQYQQIL
B03	FSFVQYQQILSDLNV
B04	YQQILSDLNVPVSIV
B05	SDLNVPVSIVFIGFL
B06	PVSIVFIGFLHINLS
B07	FIGFLHINLSAIELM
B08	HINLSAIELMSIFWK
B09	AIELMSIFWKGPMRL
B10	SIFWKGPMRLQQASL
B11	GPMRLQQASLILIAV
B12	QQASLILIAVTVTLT
B13	ILIAVTVTLTIMQIL
B14	TVTLTIMQILPQWTS
B15	IMQILPQWTSWALLV
B16	PQWTSWALLVTLALW
B17	WALLVTLALWDLFAV
B18	TLALWDLFAVLTPCG
B19	DLFAVLTPCGPLKLL
B20	LTPCGPLKLLVETAE
B21	PLKLLVETAEERGED
B22	VETAEERGEDLMPAI
B23	ERGEDLMPAIIYTGS
B24	LMPAIIYTGSASLPQ
B25	IYTGSASLPQSDSIE
B26	ASLPQSDSIESRRTS
B27	SDSIESRRTSEVKEC
B28	SRRTSEVKECTPVQD
B29	EVKECTPVQDKVKSV
B30	TPVQDKVKSVPSSGR
C01	KVKSVPSSGRFIESF
C02	PSSGRFIESFRMPSL
C03	FIESFRMPSLKSNDD
C04	RMPSLKSNDDERNIR
C05	KSNDDERNIRLGLGD
C06	ERNIRLGLGDFIFYS
C07	LGLGDFIFYSLLVGT
C08	FIFYSLLVGTASTHG
C09	LLVGTASTHGDWATT
C10	ASTHGDWATTLALFR
C11	DWATTLALFRVYLNR
C12	LALFRVYLNRSWFHS
C13	VYLNRSWFHSCSPSS
C14	SWFHSCSPSSTSKGT
C15	CSPSSTSKGTASTTN
C16	TSKGTASTTNIRHIW
C17	ASTTNIRHIWCDRYF
C18	IRHIWCDRYFSSRFA
C19	CDRYFSSRFAMSKFA
C20	SSRFAMSKFAVELNR
C21	MSKFAVELNRKQIFI
C22	
C23	DREKLQERLAKLAG
C24	HPGSVNEFDF
C25	AVNFPNPPGKGGG
C26	QEVRKYF

Figure S1. List of synthesized peptides covering the entire sequence of aspartyl protease (C7BVX5) from *Angiostrongilus cantonensis*. (F3, F4, F5, F11, F12, F13 positive controls and F9, F17 negative controls.

Entry	E-value	Score	Ident (%)	Organism	Protein name	Length
C7BVX5	-	-	-	*Angiostrongylus cantonensis*	Presenilin	415
A0A0K0DB71	0.0	1,584	77.5	*Angiostrongylus cantonensis*	Presenilin	414
A0A0D8XWV7	1.3e-132	1,005	64.2	*Dictyocaulus viviparus*	Presenilin	315
A0A016U204	5.4e-110	866	45.7	*Ancylostoma ceylanicum*	Presenilin	425
A0A016TZZ8	9.6e-108	852	45.6	*Ancylostoma ceylanicum*	Presenilin	435
A0A0R3PEK8	1.0e-128	972	79.8	*Angiostrongylus costaricensis*	Presenilin	409
G0N7M3	6.5e-118	904	77.0	*Caenorhabditis brenneri*	Presenilin	444

Figure S2. Identity of the *A. cantonensis* aspartyl protease (C78VXS) used in our study with major other proteins deposited in data bank.

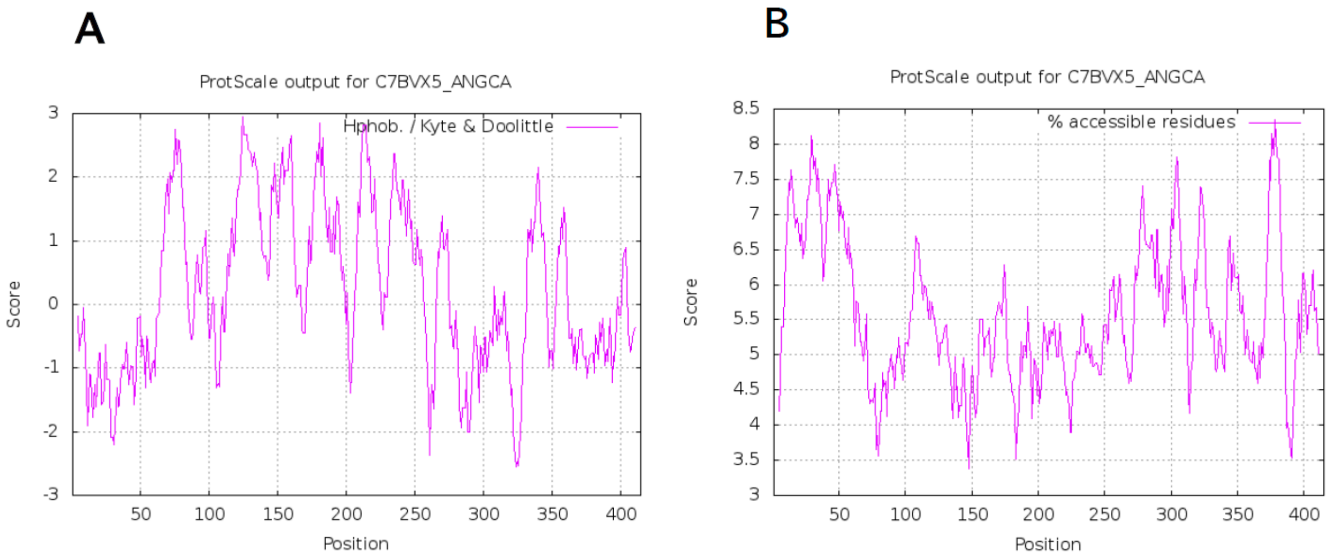

Figure S3. Hydropathycyty (**A**) and amino acid accessibility (**B**) of aminoácids of the A. cantonensis aspartyl protease.

```
1: ------IHGRFVMEDGSGSNTDLKQEVKTVNDETASKSKERTI-----STTARNSKAASTNGISEKVKRLYGPTDMVKVF 69
2: -------------------------------------------------------------------------------- 0
3: ------------MEDGSGSNTDLKQEVKTVNDETASKSKERTI-----STTARNSKAASTNGISEKVKRLYGPTDMVKVF 63
4: MSSSTVCTEQELVLSGIDTSEE--SP-ITMANEARSRSGNENVNGSRPTTSAEVATTSAEIASSSRKEKNYGPQDMVKVF 77
5: MS-SSKSIGKTLYVKGIDARHD--EE-DAITGEKKRRGENAAAAGSS-------RAKAKKSTSHRQHEKNYGPKDMVKVF 69
6: --P-------------SCTS-------QPGTQEHPA-------------TVRRTSTLGSGENEEEEAELKYGASHVINLF 45
7: MPY-------------SREP-------QDGGGNTSASSETHTT---YGTNLISNRSEPEEENQVEEAELKYGASHVIHLF 57

                       PSAg1                            PSAg2           PSAg3
1: VPVSITMFIVVTCVRNFDIYHGVNLIPTPYVIYNEPAADPGTKLLHAVANAATFLVVVAISTFALLCLFYYKFYCFLTGF 149
2: ------------------------------MIYTEPSDDAVTELLHAIINASTFLLLVVISTFLLLFLFYYKFYCLLHGF 50
3: VPVSITMFIVVTCVRNFDIYHGVNLIPTPYVIYNEPAADPGTKLLHAVANAATFLVVVAISTFTLLCLFYYKFYCTG--- 140
4: VPVSICMAIVTVITRTVEIYRQDVTIKTPYVIYHDPEAEAPTKLWHSLVNAATFLCVIVVSTFGVLALFYFKFYRCLNFF 157
5: VPVSICMTIVIICTRNVEVYQKDIILRTPYVIYHDPEAETPTKLWHSMVNAAVLLCVVVVATFGVLALFYCKCYRFLTIF 149
6: VPVSICMVMVVFTMNTVTFYSYNDGRHLLYTPFVKETDSTSEKVLMSLGNALVMLCVVIIMTVILIVFYKYRCYKVITAW 125
7: VPVSLCMALVVFTMNTITFYSQNNGRHLLYTPFVKETDSIVEKGLMSLGNALVMLSVVIVMTVLLIFFYKYKFYKLIHGW 137

                PSAg4                                  PSAg5
1: IMFSTFLLVSLFSFVQYQQILSDLNVP------------VSIVFIGFLHINLSAIELMSIFWKGPMRLQQASLILIAVTV 217
2: IMLSTFMLVTLFSFMQYQQILSDLNIP------------VSIVVMVFIHLNIAVVQLMSIFWIGPMKVQQGSLILISVTV 118
3: ---------------YFRQILSDLNVP------------VSIVFIGFLHINLAAIELMSIFWKGPMRLQQASLILIAVTV 193
4: IMFSTFMLITTASLMQYYEVLRAINFP------------ISSATVLLLHTNMAVIGLMSIFWKGPRRVQQGTLVLMAAMV 225
5: FMFATFMLITIMTALQYHEILRAINVP------------VSAVTFLFLHLNVAVIGLMSIFWKGPMKVQQATLILMAAMV 217
6: LMVSSCLLLFLFSTIYFAVL-KSFTSCEFSFFQRSDEKAVETTASMFSLRFFLLLDSTCLTHCH--PFFQFYLIAMSALM 202
7: LIISSFLLLFLFTAIYVQEVLKSFDVS------------PSVILVLFGLGNYGVLGMMCIHWKGPLRLQQFYLITMSALM 205

           PSAg6                                        PSAg7
1: TLTIMQILPQWTSWALLVTLALWDLFAVLTPCGPLKLLVETAEERGEDLMPAIIYTGSASLPQSDSI------------- 284
2: TLTIMQILPKWTSWTLLVTLALWDLFAVLSPCGPLKLLVETAEKRGEDLMPAMIYTGIASFLQSTSSK------------ 186
3: TLTIMQILPQWTSWALLVTLALWDLFAVLTPCGPLKLLVETAEERGEDLMPAIIYSMLVLLHFHNQTQLNRVARQKLK-N 272
4: TLTIMRVLPRWTNWAMVILLAFWDLCAVLSPCGPLKILVETAEERGEDLMPAIIYTGAASIEKTESTQ------------ 293
5: TLTIMRVFPKWTSWAIVILLAFWDIFAVLSPCGPLKILVEIAEERDEELMAAIIYSTQPFFEKKKLKLTYDI---CIS-A 293
6: ALVFIKYLPEWTVWSVLAVISVWDLIAVLAPNGPLRILVETAQERNEPIFPALIYSLQGIVFPFVCVAVSGFY-FYLVFP 281
7: ALVFIKYLPEWTVWFVLFVISVWDLVAVLTPKGPLRYLVETAQERNEPIFPALIYSS-GIIYPYVLVTAVKNSEEGTEMP 384

                                              PSAg8
1: -----------ESRRTSEVKECTPVQDK----VKSVPS----SGRFIESFRMPS---------LKS-NDDERNIRLGLGD 335
2: ------------SPTSAEVEGSSPIKDK----IKT-KS----SGNTVESLRMPS---------LNS-EAEERNIRLGLGD 235
3: GIEIFDDGKPAISYKFIVPRDCTPVQDK----VKSVPS----SGRFIESFRMPS---------LKS-NDDERNIRLGLGD 334
4: G----SQTPP-------TAVDRSPTNEK----RSASRS----PLRRSGSQPIPS---------NSVSAEEESNIRLGLGD 345
5: GSATLSRTEPKQSDSLSASGEESPTK-E----RRSSRS----SVRTVESLRIPS---------FSETDEEDRNIRLGLGD 355
6: IS--------SNLPLE----------FVFS-CFEACFTAGMVPSFLVLHN-------------VVQICNISEGVKLGLGD 329
7: TTSSASSSQPSNVPKKTTKVKRIPQKVQIETNVSSNPNQGETPVARVERERQPIVNVEPVQYVRHEDYQEEKGVKLGLGD 364

   PSAg9         PSAg10         PsAg11                   PSAg12         PSAg13
1: FIFYSLLVGTASTHGDWATTLALFRVYLNRSWFHSCSPSSTSKGTASTTNIRHIWCDRYFSSRFAMSKFAVELNRKQIFI 415
2: FIFYSLLVGSASVDGDWVTTLACFVSILTGLGFTLVLLVILQKALPALPISITFGVLAYFSSRYAVSKFVLELNRKLMFL 315
3: FIFYSLLVGTASTHGDWATTLACFVSILTGLGFTLVLLVLLQKALPALPISVTFGVIAYFSSRFAMSKFAVELNRKQIFI 414
4: FIFYSLLVGNATVLADWTTIVACAVSILVGLAFTLVLLVIFQKALPALPISIAFGAIAFFSSKFVTSKYLDRLNSEQIFL 425
5: FIFYSILVGNAAVLADLTTILACFVSILVGLGFTLILLVIVQKALPALPISIAFGAITFFVSKLVTSKLIDQLNKEAIFL 435
6: FIFYSVLVGKASSYFDWNTTVACYVAILVGLCFTLLLLAVFRRALPALPISIFAGLLFYFCTRWIVTPYVSEITRRQWVY 409
7: FIFYSVLLGKASSYFDWNTTLACYVAILIGLCFTLVLLAVFKRALPALPISIFSGLIFYFCTRWIITPFVTKFTQNCLLY 444
```

Figure S4. Cross-reactivity of the six-presenilin nematode proteins deposited in the database with a higher degree of identity (45–80%) with *A. cantonensis*. BLAST was carried out in the UniProt (Universal Protein) database of the EBI (European Bioinformatics Institute), using the C7BVX5 protein from the organism "*Angiostrongylus cantonensis*" as input. (1) C7BVX5 (*Angiostrongylus cantonensis*); (2) A0A0D8XWV7 (*Dictyocaulus viviparous*); (3) A0A0K0DB71 (*Angiostrongylus cantonensis*); (4) A0A016U204 (*Ancylostoma ceylanicum*); (5) A0A0 16TZZ8 (*A. ceylanicum*); (6) A0A0R3PEK8 (*A. costaricensis*); (7) G0N7M3 (*Caenorhabditis brenneri*).

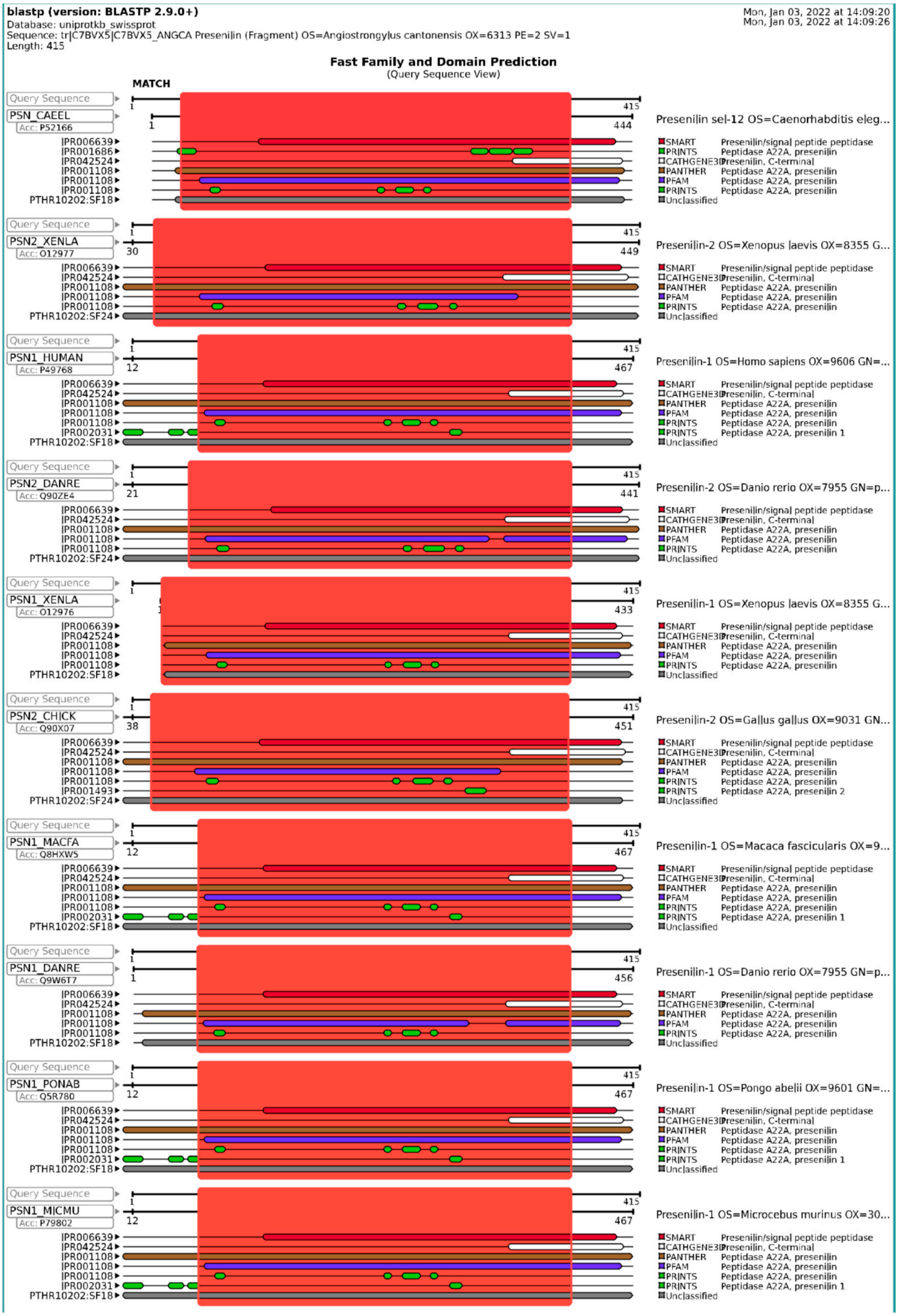

Figure S5. Multiple sequence alignment for functional prediction of AgPS.
